# An overview of patients with intertrochanteric femoral fractures treated with proximal femoral nail fixation using important criteria

**DOI:** 10.1186/s12891-024-08197-0

**Published:** 2024-12-20

**Authors:** Ümit Aygün, Eyüp Şenocak, Mehmet Fatih Aksay, Ali Can Çiçek, Orkun Halaç, Serdar Toy

**Affiliations:** 1 Faculty of Medicine, Department of Orthopaedics and Traumatology, Ağrı İbrahim Çeçen University, Ağrı, Türkiye; 2 Faculty of Medicine, Department of Orthopaedics and Traumatology, Atatürk University, Erzurum, Türkiye; 3 Orthopedics and Traumatology Clinic, Ağrı Training and Research Hospital, Ağrı, Türkiye; 4 Department of Orthopaedics and Traumatology Clinic, Başakşehir Çam ve Sakura City Hospital, İstanbul, Türkiye

**Keywords:** Intertrochanteric femoral fracture, Tip-apex distance, Postoperative reduction classification, Cleveland zone, Radiographic union scale for the hip, Functional scores

## Abstract

**Background:**

This study aimed to assess important criteria, including osteoporosis, fracture type, implant position within the bone, fracture reduction, and radiographic union, in patients with intertrochanteric femoral fractures treated with proximal femoral nail (PFN) fixation and to show their effect on clinical outcomes.

**Methods:**

PFN fixation was applied in 73 patients (41 females, 32 males; mean age: 64.5 ± 6.2). The T score, fracture type according to the AO Foundation and Orthopedic Trauma Association (AO/OTA), implant-related complications (IRCs), Harris hip score (HHS), Jensen social function (JSF) score, and Parker-Palmer mobility score (PPMS), postoperative reduction classification, screw position according to the Cleveland zone, Radiographic Union Scale for the Hip (RUSH) score, and tip-apex distance (TAD) were recorded.

**Results:**

Most screws were in central-central (*n* = 42) and inferior-central (*n* = 11) positions. IRCs were seen mostly in cases of screws placed in peripheral zones (*n* = 10) and were not observed in almost any patient with a TAD ≤ 25 mm (*n* = 52) *(p <* 0.001). Most of the patients with acceptable or good fracture reduction did not have IRCs (*n* = 11 and 50, respectively) (*p* < 0.001). Half of type 3A1 (*n* = 13) and most type 3A2 (*n* = 20) fractures showed radiographic union at 3 months, and most type 3A3 (*n* = 9) fractures showed radiographic union at 5 months (*p* < 0.05). At 12 months, type 3A2 fractures had the highest HHS (79.2 ± 5.3) and PPMS (3.9 ± 1.5), while type 3A3 fractures had the lowest HHS (70.3 ± 4.6) and PPMS (0.6 ± 2.8) (*p* < 0.05). At 12 and 24 months, type 3A3 fractures had higher JSF scores (2.8 ± 0.4 and 3.5 ± 0.5, respectively); at 3 months, type 3A1 fractures had higher JSF scores (3.1 ± 0.3) (*p* < 0.05). The radiographic bone union time was prolonged in patients with a T score ≤ -2.5 standard deviation (SD) (*p* < 0.05). The HHS and PPMS increased while the RUSH score, considered as the radiographic union, was going towards the 4th month and decreased after the 4th month (*p* < 0.05).

**Conclusions:**

Considering the criteria (osteoporosis, fracture type, implant position within the bone, fracture reduction, and radiographic union) examined in this study, satisfactory results can be obtained with PFN fixation in the treatment of patients with intertrochanteric femoral fractures.

## Background

Hip fractures cause functional problems. More than half of these patients are unable to regain their previous level of mobility [1]. These fractures cause serious issues for millions of people, placing a significant strain on healthcare systems [2]. 50% of hip fractures are intertrochanteric femoral fractures, and the mortality rate within a year is 15–20% [3]. Numerous morbidities, including diabetes, heart disease, hypertension, lung disease, and poor general health, are present in this group of patients [4], which is why the acknowledged standard of care for this kind of fracture is to achieve early mobility with a surgical procedure that will provide anatomical alignment and stable fixation.

Biomechanical studies have led to the development of intramedullary implants suitable for treating trochanteric fractures due to their load-bearing capability and good mechanical properties [5]. Functional results vary according to factors ranging from the position of the implant in the femoral neck to the course of the fracture and patient-related data. Achieving osteosynthesis by correctly positioning the implant can lead to good union and minimize mechanical complications [6]. Evaluating the criteria for intertrochanteric femoral fractures treated with PFN fixation may help clinicians identify key factors influencing treatment outcomes, such as fracture reduction quality, implant positioning, and biomechanical stability. This may ensure optimal patient recovery, reduce complications, and enhance functional outcomes [7, 8]. By defining and analyzing the criteria mentioned, this study provides evidence-based insights into the best proximal femoral nail (PFN) fixation practices. It may inform surgeons on selecting the most appropriate surgical techniques, avoiding pitfalls (e.g., malalignment or implant failure), and tailoring treatment to patient-specific anatomical or fracture characteristics.

The aim of this study was to assess important criteria, including osteoporosis, fracture type, implant position within the bone, fracture reduction, and radiographic union, in patients with intertrochanteric femoral fractures treated with PFN fixation and to show their effect on clinical outcomes.

## Materials and methods

This study was designed as a retrospective cohort study and was approved by a local ethics committee. We conducted the research in compliance with the principles of the Declaration of Helsinki, and informed consent was obtained from the patients.

Between January 2016 and November 2020, 106 patients were admitted to our hospital with a diagnosis of intertrochanteric femoral fracture. Among them, patients with unilateral intertrochanteric fractures, patients treated with the same type of implant, and patients who completed all regular follow-ups until the last visit in the second year after surgery in accordance with the study design were included in the study. Those with pathological fractures, those with multiple fractures, those who had previously undergone orthopedic surgery for an extremity fracture, those who had postoperative cerebrovascular or cardiopulmonary disorders that impair patient function, and those who died were excluded from the study. Therefore, 73 patients constituted the study group.

Various data, including age, sex, additional disease, T score for osteoporosis, intertrochanteric fracture classification according to the AO Foundation and Orthopedic Trauma Association (AO/OTA), implant-related complications (IRCs), were recorded using the hospital archive and patient controls. Harris hip score (HHS) [9], Jensen social function (JSF) score [10], and Parker-Palmer mobility score (PPMS) [11] in the preoperative (before fracture, with the information received from the patients and their relatives) and postoperative periods, were evaluated. A similar rehabilitation program was used with all of the patients. The postoperative reduction classification [12], screw position according to the Cleveland zone [13], tip-apex distance (TAD) [14], and the Radiographic Union Scale for the Hip (RUSH) score [15] were recorded using postoperative radiographs. All patients underwent X-ray examinations according to similar protocols, and radiographic evaluations were performed by 3 orthopedic surgeons twice with an interval of one month in a blinded manner.

The AO-OTA classification was divided into 3 groups: 3A1, 3A2, and 3A3. Additional diseases included diabetes mellitus (DM), cardiac – hypertension, pulmonary diseases, and neurologic diseases. Patients with a T score ≤ -2.5 standard deviation (SD) were considered to have osteoporosis. The IRCs recorded were cut-out, migration, varus malalignment, and aseptic loosening.

**HHS**: The components of this score are pain, function, range of motion, and deformity. The function is subdivided into activities of daily living and gait. The score was categorized as follows: < 70, poor; 70–79, fair; 80–89, good; and 90–100, excellent. The HHS was evaluated preoperatively and at 6 and 12 months postoperatively. **JSF score**: The JSF score is graded as follows: (1) independent; (2) slightly dependent; (3) moderately dependent; and (4) totally dependent. **PPMS**: The parameters of the PPMS are as follows: (a) able to walk inside the house; (b) able to walk outside the house; and (c) able to go shopping, to a restaurant, or to visit family. The parameters are scored as follows: 3 points, able to perform with no difficulty; 2 points, able to perform alone with an assistive device; 1 point, able to perform with help from another person; and 0 points, not able to perform at all. The total score ranges from 0 to 9. The JSF score and PPMS were evaluated preoperatively and at 3, 6, 12, and 24 months postoperatively.

### Postoperative reduction classification

The categories for postoperative reduction are as follows: 1) Alignment: (a) anterior-posterior view: normal or mildly valgus neck-shaft angle, (b) lateral view: less than 20° of angulation; and 2) Displacement: (a) anterior-posterior view: less than 4 mm of displacement of any fragments, (b) lateral view: less than 4 mm of displacement of any fragments. Reduction quality was considered good if both criteria were met, acceptable if only one criterion was met, and poor if neither criterion was met.

### Cleveland zone

On the anterior-posterior radiograph, the femoral head is segmented into superior, central, and inferior thirds, and anterior, central, and posterior thirds on the lateral radiograph. As a result, there are a total of nine different zones where the screw can be found.

### RUSH score

In Sect. 1, the overall subjective impression of healing is recorded as “healed” or “not healed.” In Sect. 2, four components are evaluated: the cortex is evaluated for bridging (1) and disappearance of the fracture line (2), and the trabecular appearance is evaluated for consolidation (3) and disappearance of the fracture line (4). A score of ≥ 20 was considered to indicate union.

### TAD

The TAD was computed as the sum of the distances measured on the anteroposterior and lateral views from the screw’s tip to the apex of the femoral head.

### Statistical analysis

SPSS (Statistical Package for Social Sciences) version 21.0 (IBM, Armonk, NY, USA) was used in the analysis. Relationships between categorical variables were analyzed by chi-square test. Relationships between numerical variables were analyzed by Pearson correlation analysis. The conformity of continuous variables to normal distribution was examined with the Kolmogorov-Smirnov test. The difference between numerical variables (T-score, TAD, HHS, PPMS, JSF, and RUSH) and 2-group categorical variables (AO/OTA classification, IRCs, screw position according to Cleveland zones, postoperative reduction classification) was analyzed using the t-test, and the difference between numerical variables and three or more group categorical variables was analyzed using the ANOVA test. Numerical variables are expressed as mean ± SD. The statistical significance level was set at *p* < 0.05.

## Results

The mean follow-up time of the 73 patients (41 female, 32 male) aged between 50 and 80 (mean: 64.5 ± 6.2) was 26.4 (range: 22–31) months. Most fractures were type 3A2 fractures (*n* = 36), most patients with comorbidities had more than one disease (*n* = 35), and most IRCs were cut-out (*n* = 6) (Table 1).

**Table 1:** Demographic and clinical characteristics of patients who underwent PFN fixation

		PFN	
		N	%
Age (Years), Mean ± Sd	64.5 ± 6.2	73	100
Sex	Female	41	56.1
	Male	32	43.8
AO-OTA	3A1	26	35.6
	3A2	36	49.3
	3A3	11	15.0
Additional illness	Only one	18	33.9
	> 1	35	66.0
IRCs	Cut - out	6	50.0
	Varus malalignment	2	16.6
	Migration	2	16.6
	Aseptic loosening	2	16.6

N; Number Sd; Standard deviation PFN; Proksimal Femoral Nail AO-OTA; AO Foundation and Orthopedic Trauma Association IRCs; Implant-related complications

**Fig. 1:**
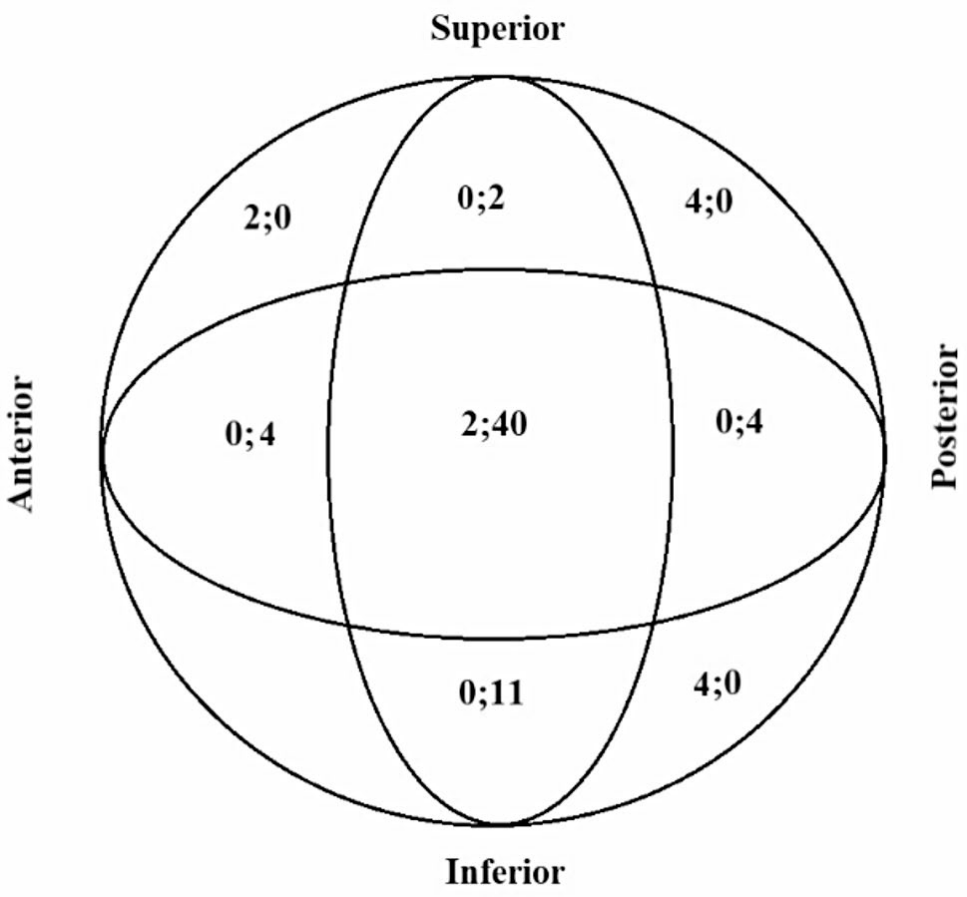
Screw position according to the Cleveland zone system. The first number represents the number of patients with IRCs, and the second number represents the number of patients without IRCs

**Fig. 2:**
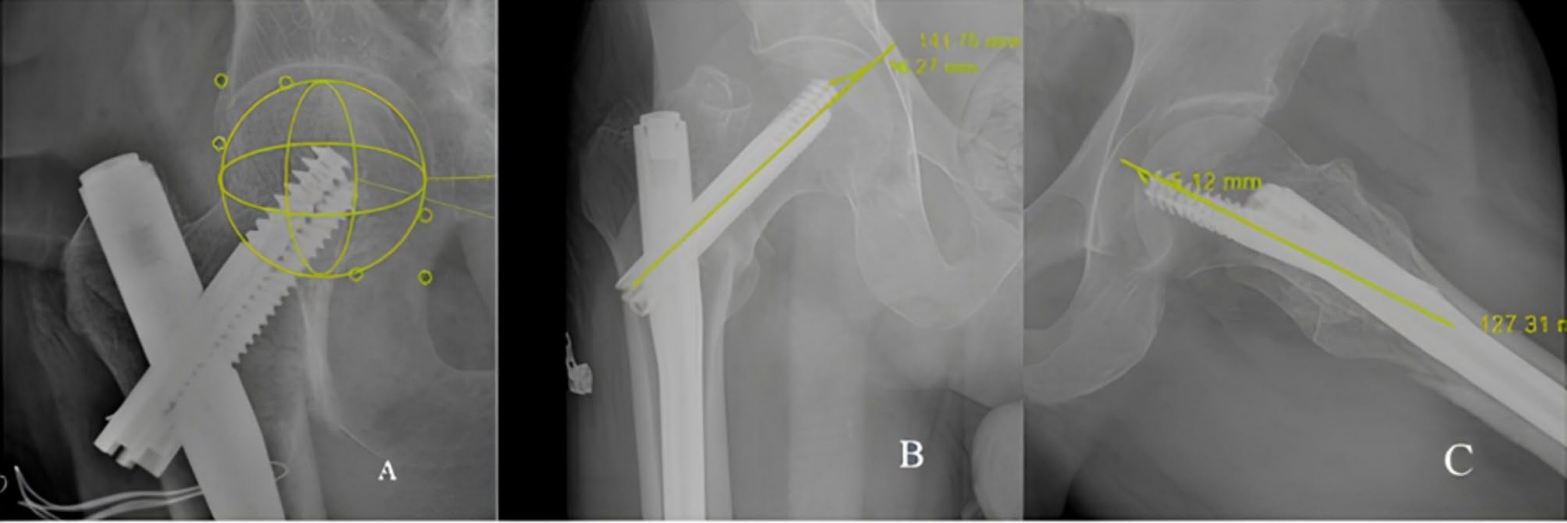
Examples of radiographic evaluation of PFN fixation. (**A**) Cleveland zone. (**B**) AP X-ray; TAD. (**C**) Lateral X-ray; TAD

According to the Cleveland system, most of the screws were in a central-central (*n* = 42) or inferior-central (*n* = 11) position, and IRCs were seen mostly in cases of screws placed in peripheral zones (*n* = 10) rather than centrally (*n* = 2) (*p* < 0.001) (Figs. 1 and 2A).

**Fig. 3:**
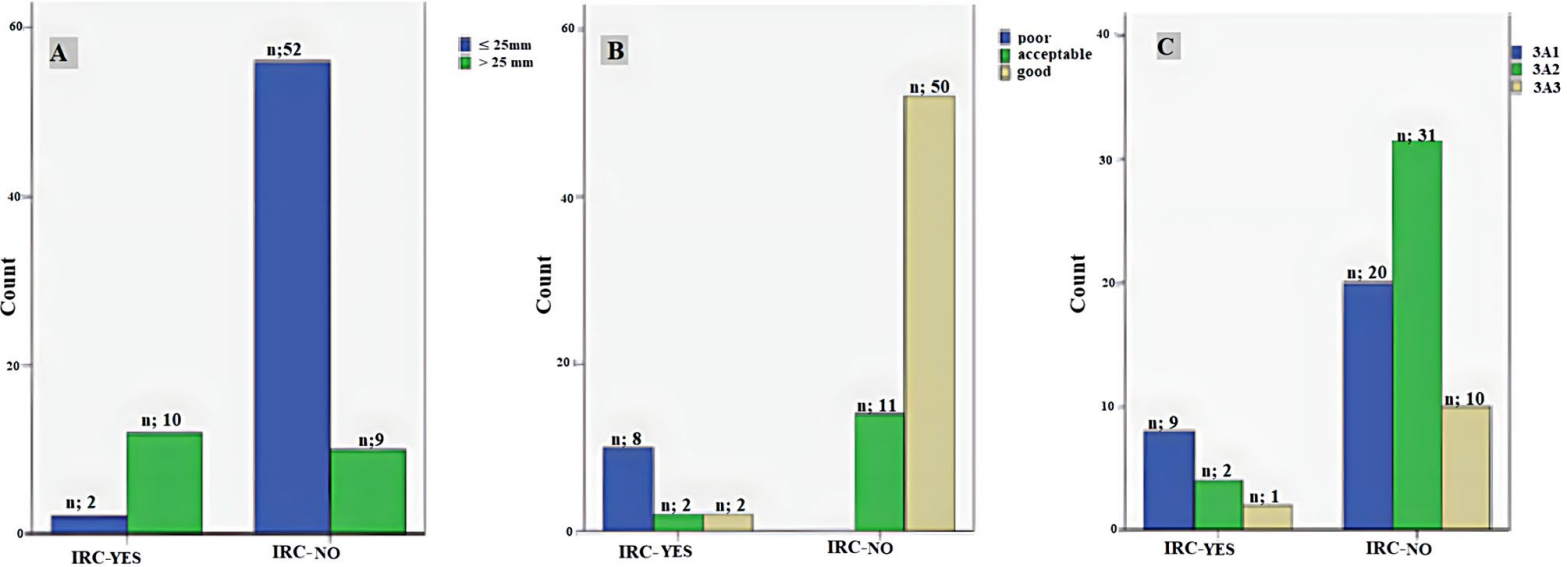
Relationship of the TAD (**A**), postoperative reduction classification (**B**), and AO-OTA classification (**C**) with IRCs

The mean TAD was 21.0 ± 4.5 mm (range: 15–38 mm). IRCs were observed in most patients with a TAD > 25 mm (*n* = 10, 13.6%), while complications were not observed in almost any patient with a TAD ≤ 25 mm (*n* = 52, 71.2%) (*p* < 0.001) (Figs. 2B-C and 3A). According to the postoperative reduction classification, most of the patients with acceptable or good fracture reduction did not have IRCs (*n* = 11 and *n* = 50, 15.0% and 68.4%, respectively), while IRCs were observed in all patients with poor fracture reduction (*n* = 8, 10.9%) (*p* < 0.001). There was no significant relationship between fracture type and IRCs (*p* > 0.05) (Fig. 3B-C).

We obtained the RUSH score accepted as an indicator of the union at an average of 3.7 ± 0.8 months (range: 3–5 months). When the AO fracture type was evaluated together with the RUSH score, half of type 3A1 (*n* = 13) and most type 3A2 (*n* = 20) fractures showed radiographic union at 3 months, and most type 3A3 (*n* = 9) fractures showed radiographic union at 5 months (*p* < 0.05). At 12 months, type 3A2 fractures had the highest HHS and PPMS, and type 3A3 fractures had the lowest HHS and PPMS (*p* < 0.05). At 12 and 24 months, type 3A3 fractures had higher JSF scores; at 3 months, type 3A1 fractures had higher JSF scores (*p* < 0.05) (Table 2).

**Table 2:** Association of AO-OTA intertrochanteric fracture classification with RUSH score, HHS, JSF score, and PPMS

		AO - OTA			
	Month	3A1	3A2	3A3	*P*
RUSH, n	3.	13	20	2	0.011*
	4.	9	11	0	
	5.	4	5	9	
HHS	6.	73.5 ± 6.7	76.5 ± 5.3	72.6 ± 4.6	0.083
	12.	75.7 ± 6.8	79.2 ± 5.3	70.3 ± 4.6	0.036*
JSF score	3.	3.1 ± 0.3	2.6 ± 0.4	2.9 ± 0.0	0.005*
	6.	2.6 ± 0.5	2.5 ± 0.6	2.8 ± 0.4	0.406
	12.	2.2 ± 0.7	1.9 ± 0.5	2.8 ± 0.4	0.006*
	24.	2.0 ± 0.8	1.8 ± 0.7	3.5 ± 0.5	0.001*
PMS	3.	3.6 ± 1.3	3.8 ± 1.0	0.8 ± 2.5	0.091
	6.	3.7 ± 1.2	3.6 ± 1.1	0.9 ± 1.9	0.163
	12.	3.5 ± 1.8	3.9 ± 1.5	0.6 ± 2.8	0.029*
	24.	3.5 ± 2.1	3.6 ± 1.8	0.8 ± 2.1	0.126

*Significance RUSH; Radiographic Union Scale for the Hip *(considered as radiographic union)* HHS; Harris Hip Score JSF; Jensen social function PPMS; Parker-Palmer mobility score n; number

The RUSH score did not have a significant relationship with the presence of additional diseases (*p* > 0.05). The mean T score was − 2.4 ± 0.5 SD (range: -1.7 to -3.8), which can be considered to be within the osteoporosis range. The radiographic union time was prolonged in patients with a T score ≤ -2.5 SD (*p* < 0.05). The HHS and PPMS increased while the RUSH score considered as the radiographic union was going towards the 4th month and decreased after the 4th month, i.e., as the radiographic union time increased (*p* < 0.05). The JSF scores at 12 and 24 months progressed well, while the RUSH score required for the radiographic union was going towards the 4th month, and similarly, these scores worsened after the 4th month (*p* < 0.05) (Table 3). The preoperative and postoperative HHS, JSF score, and PPMS were found to be positively related, but the scores did not reach the preoperative level during the evaluation period (Table 4).

**Table 3:** Relationship of RUSH score with T score, additional diseases, HHS, JSF score, and PPMS

			RUSH			*P*
			3.mth	4.mth	5.mth	
T score, n	≤ -2.5 SD		7	5	16	0.001*
	> -2.5 SD		28	15	2	
Additional diseases, n	Only one		11	5	2	0.442
	> 1		19	4	12	
HHS	mth	6.	75.9 ± 5.3	76.9 ± 6.2	69.5 ± 4.3	0.005*
		12.	78.0 ± 5.8	80.0 ± 5.7	71.5 ± 3.8	0.002*
JSF score	mth	3.	2.7 ± 0.4	2.9 ± 0.5	3.1 ± 0.3	0.128
		6.	2.5 ± 0.5	2.6 ± 0.6	2.8 ± 0.3	0.074
		12.	2.0 ± 0.6	1.8 ± 0.6	2.7 ± 0.4	0.004*
		24.	2.1 ± 0.8	1.7 ± 0.6	3.1 ± 0.7	0.001*
PPMS	mth	3.	3.7 ± 1.0	4.3 ± 1.0	2.5 ± 0.7	0.001*
		6.	3.6 ± 0.9	4.3 ± 1.1	2.4 ± 0.8	0.001*
		12.	3.7 ± 1.4	4.5 ± 1.6	1.7 ± 0.6	0.001*
		24.	4.0 ± 1.7	5.4 ± 1.7	2.2 ± 0.7	0.001*

*Significance n; number mth; month SD; standard deviation

**Table 4:** Comparison of preoperative and postoperative HHS, JSF score, and PPMS

	Month		Preoperative HHS
Postoperative HHS	6.	r	0.960*
	12.		0.968*
			Preoperative JSF score
Postoperative JSF score	3.	r	0.470*
	6.		0.569*
	12.		0.817*
	24.		0.796*
			Preoperative PPMS
Postoperative PPMS	3.	r	0.929*
	6.		0.869*
	12.		0.927*
	24.		0.953*

* r; correlation

## Discussion

One of the components affecting stability following fracture treatment is reduction quality [16]. Various criteria for evaluating the reduction quality of trochanteric fractures have been documented in the literature [17]. The criteria developed by Baumgaertner et al. [14]. are widely used. It has been shown that poor reduction of these fractures leads to complications [18]. Baumgaertner and Solberg reported that with poor reduction, fractures were more than three times more likely to progress to cut-out [19]. Another study found an association between poor reduction and implant failure. Poor reduction quality and loss of posteromedial support have been shown to cause implant failure in unstable intertrochanteric femoral fractures [20]. In a study of 127 patients, researchers used the Baumgaertner reduction quality criteria (BRQC), and they showed that the rates of mechanical complications were 8.6% (good BRQC), 21.7% (acceptable BRQC), and 88.9% (poor BRQC) [21].

Cleveland et al. [13]. showed that the highest rates of cut-out occurred in the posterior-inferior zone and the anterior-superior zone. The cut-out rate in either of these two peripheral zones was significantly higher than in the center zone. There were no problems in terms of cut-outs in the remaining zones. Another study showed that the highest rate (85.7%) of poor outcomes occurred in the superior-central Cleveland zone [21]. Because of the physiological anteversion of the femoral neck, central zones are not exposed to rotational forces, whereas peripheral zones are exposed to rotational forces due to biomechanical reasons [22].

In this study, almost no mechanical complications were observed among screws placed in the central Cleveland zones, while mechanical problems were observed among screws placed in the peripheral zones. While most of the fractures with mechanical complications were classified as having a poor reduction, almost all fractures with acceptable or good reduction showed no problems. This study shows that screw position and postoperative reduction classification criteria are important in terms of IRCs, in accordance with the literature [13, 18, 20].

The fact that internal fixation for intertrochanteric femoral fractures can cause problems such as cut-out and loss of reduction at the fracture line in elderly patients has directed some surgeons toward hemiarthroplasty [23]. Additionally, cut-out complication rates are lower with newer intramedullary nail designs [24]. Biomechanical studies have shown superior stabilization of the proximal fragment of the femur with blades vs. screws [25]. Research has also shown that placing two lag screws in the femoral head provides better protection against rotation, thus reducing the risk of cut-out. Additionally, an anatomical reduction is important, especially regarding the avoidance of varus malalignment. On the anterior to posterior (AP) view, the inferior screw should be as close as possible to the inferior femoral cortex, and both screws should be close to the central part of the femoral head [22, 26]. Placing the implant tips in the subchondral region of the femoral head has been shown to cause fewer complications [14]. In a study, neither loss of reduction nor cut-out was observed at the end of the one-year follow-up period, even if the TAD was > 25 mm for screws placed 5 mm from the subchondral region, with good bone quality [22]. In this study, the absence of complications in almost half of the patients with a TAD > 25 mm can be explained by the double-screw implant placed in the femoral neck and close patient follow-up; however, this situation suggested that other factors, such as surgical technique, should be considered.

Emphasizing the importance of the TAD, the criteria proposed by Baumgaertner et al. [14]. have guided some studies to avoid implant failure [27, 28]. Their study showed that no cut-out occurred when the TAD was less than 25 mm [14]. In Geller’s study of 82 pertrochanteric fractures, it was concluded that the surgeon should aim for a TAD < 25 mm when placing the intramedullary device [28]. However, it was emphasized that the TAD should be < 15 mm in a study using a 135° dynamic hip screw device [27]. In a study of 127 patients, 26 patients developed mechanical complications such as implant failure, varus displacement, and excessive lateral migration of the blade; among them, the mean TAD was 26.7 mm, compared with 22.9 mm among the 101 patients who achieved union uneventfully [21]. In the present study, most of the patients without IRCs had a TAD of ≤ 25 mm.

Implant failure is a common cause of mortality and morbidity in patients with pertrochanteric fractures [29]. Difficulties in intraoperative reduction may be encountered in elderly patients with Evans-Jensen type III or above trochanteric fractures due to the lack of support of the femoral neck, mechanical factors such as anti-rotation, and problems such as screw loosening and cut-out in cases of severe osteoporosis. Studies have shown a failure rate of 7.1–12.5% for the use of proximal femoral nails in the treatment of intertrochanteric fractures [30, 31]. The incidence of AO/OTA type 31-A3 fractures is relatively low, but the incidence of implant failure in AO/OTA type 31-A3 fractures is relatively high compared with that in AO/OTA type 31-A1 and A2 fractures [18, 27]. A randomized controlled study showed that the mean time to radiographic union (RUSH score) of intertrochanteric fractures treated with intramedullary implants increased as the AO classification degree increased and that there was no statistically significant association between the fracture type (based on the AO classification) and final functional outcome [32]. In this study, half of type 3A1 and most type 3A2 fractures showed radiographic bone union at 3 months, and most type 3A3 fractures showed radiographic union at 5 months. The fact that type 3A2 fractures showed the highest PPMS and HHS at the end of one year while type 3A3 fractures showed the lowest PPMS and HHS, and that high JSF scores at 12 and 24 months in type 3A3 fractures will add a different perspective to the literature on the possibility of an inverse relationship between fracture type and patient function. We think that the higher JSF score of type 3A1 fractures in the 3rd month is related to the union (RUSH score) of only half of these types of fractures in the 3rd month and restricted movement. Although the postoperative scores showed a positive relationship with the preoperative scores, the fact that they never reached the preoperative levels indicates that this type of fracture should be handled according to the consideration of many aspects, such as surgical technique, physiotherapy, and general patient condition, especially in geriatric patients.

The prevalence of osteoporosis has been steadily rising as the population ages [33]. It has been shown that osteoporosis contributes to adverse outcomes in the treatment of pertrochanteric fractures [34]. The bone union time is an important criterion for early weight-bearing and mobilization. However, one study found no significant difference in the fracture union time according to the T score [32]. One of the important points in treating this type of fracture is stable fixation. However, the fact that most of these patients are of advanced age and have osteoporosis has a great impact on implant-related complications and morbidity [6, 23]. Due to biomechanical properties such as a shorter lever arm, intramedullary nails are more stable in this region than other implants. It is known that failure to achieve early weight-bearing is an important point, particularly in this type of fracture, which is common among elderly and osteoporotic patients [35].

In this study, the fact that most of the patients with a T score ≤ -2.5 SD achieved radiographic union in 5 months should be a warning regarding possible complications. Clinical factors of patients may have contributed to this finding [23, 34]. In this study, these factors were also reflected in the mobility and social functions of the patients. We attribute the worsening of the HHS, PPMS, and 12th and 24th month JSF score after 4 months to increased radiographic union time, prolongation of the immobilization period, and the related physiological and psychosocial effects on the patient.

The limitations of this study include its single-center and retrospective nature, the fact that all surgeries were performed by different surgeons, and the inability to compare different implant types used for osteosynthesis in treating this type of fracture. Prospective studies can be considered to provide more data control and reduce effects such as selection bias. If possible, more detailed imaging methods and evaluations in different age groups can guide the generalizability of the study. The surgical procedures performed by more than one surgeon in the study can be considered as an element that increases the generalizability of the results. However, it is important to consider the potential impact of this situation on critical parameters, especially the quality of reduction and screw placement. Blinded methods can be used in the evaluation processes to examine the variability between surgeons in more detail in multicenter studies. However, this study is important in representing the elderly population regarding the evaluated parameters.

## Conclusion

There is still a lack of prognostic indicators for functional outcomes in patients with intertrochanteric fractures. It is crucial to identify the variables that will affect long-term functional outcomes in these patients. Although the study amalgamates multiple assessment systems, its implications are substantial. Combining these systems enables a more comprehensive understanding of fracture treatment outcomes, highlighting strengths and gaps in current methodologies. The findings can promote the development of a unified assessment framework tailored for clinical application, leading to improved decision-making during surgical planning. By identifying inconsistencies or redundancies in existing assessment tools, the study can guide refinements in protocols, enhancing their relevance and reliability for treating intertrochanteric fractures. Effective use of intraoperative fluoroscopy for optimal screw placement, careful analysis of existing classification systems in the evaluation of reduction, and attention to intraoperative TAD calculation are simple methods applicable to surgeons for treatment success. This study will add a different perspective to the literature in terms of comparing many parameters with patient function in the treatment of intertrochanteric femoral fractures.

## Data Availability

The datasets used and analyzed during the current study are available from the corresponding author on reasonable request.

## Abbreviations

PFN: Proximal femoral nail
AO/OTA: AO Foundation and Orthopedic Trauma Association
IRCs: Implant-related complications
HHS: Harris hip score
JSF: Jensen social function
PPMS: Parker-Palmer mobility score
TAD: Tip-apex distance
RUSH: Radiographic Union Scale for the Hip
DM: Diabetes mellitus
SD: Standard deviation
SPSS: Statistical Package for Social Sciences
BRQC: Baumgaertner reduction quality criteria
AP: Anterior to posterior
